# Association of Secretor Status with Enteropathy and Growth among Children in Bangladesh Aged 1–24 Months

**DOI:** 10.4269/ajtmh.22-0183

**Published:** 2022-07-11

**Authors:** Parag Palit, Mondar Maruf Moin Ahmed, Md Amran Gazi, Md Ahshanul Haque, Md Ashraful Alam, Rashidul Haque, Mustafa Mahfuz, Tahmeed Ahmed

**Affiliations:** ^1^ Nutrition and Clinical Services Division, International Centre for Diarrhoeal Diseases Research, Dhaka, Bangladesh (icddr,b);; ^2^ Emerging Infections and Parasitology Laboratory, International Centre for Diarrhoeal Diseases Research, Dhaka, Bangladesh (icddr,b)

## Abstract

Secretor status refers to the ability of an individual to secrete blood group antigens into body fluids and onto the different epithelial surfaces. Concurrent findings have demonstrated an association of the secretor status of children with susceptibility to a plethora of enteropathogens. We aimed to determine a possible association of secretor status of children with childhood enteropathy, an important causal factor for childhood growth failure. Participants of the Malnutrition and Enteric Disease (MAL-ED) birth cohort study from the Bangladesh site were enrolled along with their mothers. Saliva was analyzed for determining blood groups and secretor status of the children and their mothers by using an in-house ELISA. Approximately 59% of children and 65% of mothers were found to be secretor positive. Secretor-positive children were found to have a significantly positive association with alpha-1-antitrypsin (β-coefficient: 0.11, 95% CI: 0.07, 0.21, *P* < 0.01) and with environmental enteric dysfunction score (β-coefficient: 0.32, 95% CI: 0.29, 0.65, *P* = 0.05). However, despite a negative effect size, secretor-positive children did not show any statistical significance with length-for-age and weight-for-age *z* scores (LAZ and WAZ), respectively. Our findings indicate toward the genetic factor of secretor status of children being associated with childhood growth faltering, through increased susceptibility to distinct enteropathogens and the consequent development of enteric inflammation and enteropathy among children. However, these findings are only applicable in Bangladeshi settings and thus need to be validated in several other similar settings, to establish a possible relationship between the secretor status of children with enteropathy and resulting childhood growth failure.

## INTRODUCTION

Environmental enteric dysfunction (EED) refers to the histological alterations of the architecture of the intestine that is usually specified by the increase in the depth of crypts, infiltration of lymphocytes, and decreased surface area of the intestinal mucosa.^1^ Continual immune activation as a result of chronic exposure to diarrheal enteropathogens, and enteric inflammation raised permeability of the intestine are the distinctive indications of EED.^2^^,^^3^ However, a phenomenon known as dysbiosis that indicates toward immaturity of the gut microbiome and depletion of age-specific beneficial members of the gut microbiota such as *Bifidobacterium infantis* during the first 6 months of life and *Prevotella copri* during the postweaning period have been reported to contribute to enteric inflammation and growth retardation, independent of pathogen carriage.^4^ Nevertheless, early childhood growth failure has been attributed to the chronic exposure to diarrheal enteropathogens and consequent development of the features of EED.^1^ Concurrent reports from studies carried out in the settings of the MAL-ED study have shown that asymptomatic infection by diarrheal enteropathogens such as *Campylobacter jejuni/coli*, EAEC, enterotoxigenic *Bacteroides fragilis* contribute to enteric inflammation.^5^^–^^7^

Upper gastrointestinal (GI) endoscopy is widely regarded as the gold standard for the diagnosis of EED.^8^ Although a few studies have used upper GI endoscopy to study the pathophysiology of EED,^9^^,^^10^ the invasive nature of the procedure and the requirement of specialized clinical settings and expertise renders it infeasible to be carried out among children in resource-limited settings.^8^ Concurrent findings show that several fecal biomarkers, namely myeloperoxidase (MPO; indicates intestinal inflammation), neopterin (NEO; indicates intestinal inflammation), and alpha-1 antitrypsin (AAT; indicates loss of enteric proteins and intestinal permeability), are associated with EED and thus have been proposed as noninvasive alternatives for the diagnosis of EED.^11^^–^^13^

Secretor status refers to the ability or inability of an individual to secrete histo-blood group antigens into body fluids (such as saliva and breast milk) and/or onto the different epithelial surfaces. The fucosyltransferase-2 (FUT-2) gene that encodes the FUT-2 or the secretor enzyme allows for secretion of the histo-blood group antigens into the body fluids and/or onto the various epithelial surfaces.^14^^–^^16^ The histo-blood group antigens (ABO) are structurally defined as the immunodominant glycans and are synthesized by the glycosyltransferases, which, in turn, are encoded by the ABO gene.^17^ On the other hand, individuals who do not express the ABO antigens in body fluids and/or epithelial surfaces are called “nonsecretors”.^18^^,^^19^ The Lewis blood group system is another blood group system comprising the antigens Lewis a and b forming the groups: Lewis A, Lewis B, Lewis A+B+, and Lewis Negative.^20^ The FUT-3 gene encodes the FUT-3 or Lewis enzyme, which is essential for Lewis antigen formation. The activity of the FUT2 enzyme is also required for the production of the Lewis b (Le b) antigen.^18^ Homozygous inactivating mutations in the FUT2 gene result in the nonsecretor phenotype and so nonsecretors are usually Lewis A and secretors tend to be Lewis B. Individuals with inactive FUT3 enzyme do not express Lewis a and b, and are termed Lewis-negative.^21^

Concurrent findings have shown possible associations between the positive secretor status and increased susceptibility to a number of enteropathogens, including norovirus genotype GII^22^ as well as to rotavirus,^23^ *Helicobacter pylori*,^24^^,^^25^ and *C. jejuni/coli*.^26^ Individuals who are secretors present their blood group antigens onto the epithelial surface of the intestinal mucosa, which serve as attachment sites for these enteropathogens.^27^^–^^29^ However, potential links between the secretor status and enteropathy in children have not been investigated till date. In this study, we have attempted to establish a possible association between the secretor status and enteropathy among young children in Bangladesh, to elucidate genetic cues for childhood enteric inflammation. For our study, we have used the settings of the MAL-ED birth cohort study and have enrolled the children who had been previously enrolled in the MAL-ED study at the Bangladesh site. Additionally, for the purpose of deciphering a possible intergenerational cue for childhood enteric inflammation and among the study participants we also enrolled the mothers of the study participants and also assessed the secretor status of the mothers.

## MATERIALS AND METHODS

### Study site, study participants, and ethics statement.

This present study involved the participants who were enrolled at the Bangladesh site of the MAL-ED birth cohort study. The detailed study protocol for the Bangladesh site of the MAL-ED study, conducted in the Bauniabadh slum area in Mirpur, Dhaka, has been described elsewhere.^30^ Briefly, a total of 265 participants of either sex were enrolled in the study between November 2009 and February 2012. The study participants were enrolled within 17 days of birth and followed up to 24 months of life. Among the 265 participants enrolled in the study, 210 completed the 24-month follow-up. For our current study, conducted between August and October of 2017, we reenrolled the mothers and their children who had participated in the MAL-ED birth cohort study at the Bangladesh site. Only 184 children and 152 mothers were available for our current study, because some of the children and mothers originally enrolled in the MAL-ED Bangladesh site had already migrated to distant places. The number of migrants was equally distributed across all zones of the study area. Moreover, the similar number of male and female participants had migrated during the reenrollment. Henceforth, there was minimal risk of selection bias during reenrollment. Our study was approved by the Institutional Review Board (IRB) of International Centre for Diarrhoeal Disease Research, Bangladesh (icddr,b) on August 17, 2017. The IRB of icddr,b comprises the Research Review Committee (RRC) and the Ethical Review Committee (ERC). Written informed consent was obtained from mothers or legal guardians of the children before the enrolment of the children and their mothers in our study.

### Collection of anthropometric, sociodemographic, and morbidity data.

Anthropometric measurements were made every month from enrolment till 24 months of age, using standard scales (Seca GmbH & Co. KG., Hamburg, Germany). Sociodemographic data on the child’s birth, including birth weight, anthropometric indices at birth, presence of siblings, and other maternal characteristics were collected at enrollment.^31^ A detailed account of any morbidity and child feeding practices were obtained during household visits, done twice weekly.^32^

Socioeconomic data were collected at every 6 months, starting from when the participants were 6 months old. Consequently, Water, sanitation, hygiene, Asset, Maternal education, and Income index (WAMI score), ranging from 0 to 1, an index of socioeconomic status of the households,^33^ was calculated; whereby a superior socioeconomic status was indicated by a higher WAMI score.^34^ According to the WHO guidelines, an improved sanitation facility was described as one that hygienically separated human excreta from human contact, and an improved drinking water source was defined as one that by the nature of its construction adequately protected the source from outside contamination, in particular from fecal matter.^35^ Treatment of drinking water was defined as filtering, boiling or addition of bleach.^36^

### Collection of biological specimens.

For our current study, saliva samples were collected from mother and child pairs using Oracol saliva collecting swabs (Malvern Medical Developments, Worcester, UK) (sponge-tipped samplers) after having their mouths rinsed 3–4 times with drinking water thoroughly for about 2 minutes for proper cleaning. The samplers were handled like toothbrushes and rubbed against gums and tongue for about a minute or until soaked in saliva. The samplers were then reinserted into the Oracol tubes and quickly transported from the field site to the laboratory at icddr,b, maintaining a cold chain ensured by the use of cooler boxes with ice packs and a digital thermometer to ensure the maintenance of a steady temperature of 2–8°C inside the cooler boxes.

Previously, from November 2009 to February 2012, our community research staff collected nondiarrheal stool samples on a monthly basis from enrolment of the participant after birth until 12 months of age. From 12 months of age, stool samples were collected once after every 3 months until 24 months of age. During collection of the stool samples, no fixative was added to the stool samples, and the raw, unprocessed stool aliquots were stored in −80°C freezers before further laboratory testing.

### Determination of secretor status.

Secretor status of the children and their mothers were determined by an in-house ELISA technique, following laboratory guidelines described elsewhere.^37^^,^^38^ In brief, the saliva samples were diluted using 1X phosphate-buffered saline (PBS), incubated in water batch for 10 minutes and introduced to the wells of an empty binding ELISA plate; following which the ELISA plates were incubated overnight in a humidity chamber at 4°C. In the next day, ELISA was carried out using anti-Le a, anti-Le b, and anti-A, B, O antibodies. The ELISA plates were read at 450 nm using a plate reader and a cutoff value of 0.10 was used for the Lewis and histo-blood group antigens, as described in previous literature.^37^^,^^38^ Any value equal to or above 0.10 was designated as positive. From this data, individual blood groups and secretor statuses were determined. Individuals who were positive for only Lewis A were classified as “nonsecretors” while those testing positive for Lewis B antigens along with Lewis A or the histo-blood group antigens A, B, or O were classified as “secretors.” The secretor status of some individuals (28 children and 16 mothers) could not be determined by this method, due to the optical density values of both Lewis A and Lewis B were below the cutoff value of 0.10 and thus the secretor status of these individuals was classified as “inconclusive.”

### Determination of biomarkers of enteric inflammation and detection of enteropathogens.

Enteric inflammation was assessed by measuring the levels of MPO (Alpco, Salem, NH), NEO (GenWay Biotech, San Diego, CA), and alpha-1-anti-trypsin (Biovendor, Chandler, NC) from the monthly nondiarrheal stool samples by using quantitative commercial ELISA kits. Environmental enteric dysfunction score, ranging from 0 to 10, was calculated from the three biomarkers, as described previously,^39^ and was used as the noninvasive indicator for childhood enteropathy. Categories were assigned with the values 0 (low), 1 (medium), or 2 (high). Myeloperoxidase, NEO, and AAT values were log‐transformed before subsequent analysis. The formula used for the calculation of the EED score is as follows^13^:

EED score = 2 × AAT category + 2 × MPO category+ 1 × NEO category

On the other hand, a customized multiplex real-time polymerase chain reaction (PCR) platform involving a compartmentalized primer–probe assay system, known as TaqMan Array Cards (TAC), was used for the detection of enteropathogens.^40^^,^^41^ A cycle threshold (Ct) value of 35 was considered as the cutoff value, whereby values lower than 35 were considered to be positive for the particular enteropathogen.

### Statistical analyses.

All statistical analyses were done using STATA 13 (StataCorp LLC, College Station, TX). Descriptive characteristics were represented by either mean with SD or by frequency and percentage. Student’s *t* test or χ^2^ test was done to compare the general characteristics between the secretor and nonsecretor children, depending on the nature of the data. Multivariate generalizing estimating equation (GEE) model was used to assess the potential association between secretor status of the study participants and the enteric inflammation as indicated by the inflammatory biomarkers: MPO, NEO, and AAT as well as the EED scores. The multivariate GEE was after adjusting for relevant covariates with a *P* value ≤ 0.2 in bivariate analysis. These covariates included sex of the child, age of the child, birth weight, duration of exclusive breastfeeding, maternal age, weight-for-age *z* score (WAZ) at enrolment, WAMI, and detection of enteropathogens associated with the secretor status of children. Previous findings have shown that the detection of several enteropathogens, namely norovirus genogroup II, rotavirus, typical EPEC, ETEC, *C. jejuni/coli*, *Cryptosporidium* sp., *Giardia* sp., *Vibrio cholerae*, and *H. pylori*, was associated with the secretor status of children^37^ and so the detection of these enteropathogens was also adjusted in the final multivariate GEE model done to assess the association between secretor status of children with the enteric inflammation.

We also used a multivariate GEE model to evaluate the association between the secretor status of children with the anthropometric indices of: length-for-age *z* score (LAZ) and WAZ. The covariates that were adjusted in this multivariate GEE model included sociodemographic characteristics such as sex of the child, age of the child, birth weight, WAMI score, maternal height, maternal weight, and LAZ at enrolment. Previous studies conducted in the similar settings have shown that these sociodemographic characteristics are associated with childhood growth.^7^^,^^42^^–^^44^ Enteropathogens associated with childhood malnutrition, namely EAEC, *Campylobacter* sp., ST-ETEC, *Shigella* sp., Norovirus genogroup I, *Giardia* sp.,^45^ were also included in this multivariate GEE model constructed to evaluate association between secretor status and LAZ and WAZ of children. In all the final multivariate GEE models, associations were considered to be statistically significant only when the *P* value was less than 0.05.

## RESULTS

### Distribution of secretor status among children and mothers.

Table 1 shows the distribution of the secretor status of the children enrolled in the MAL-ED study and their mothers. One hundred and eighty-four children who were originally enrolled in the MAL-ED study at the Bangladesh site were available for our study. Among them, 108 children were found to be secretors and 48 children were found to be nonsecretors. On the other hand, we were able to collect saliva samples for 152 mothers, among whom 101 mothers were found to be secretors and 35 mothers were found to be nonsecretors. We were unable to determine the secretor status for 28 children and 16 mothers using the in-house developed ELISA-based approach and so the secretor status of these children was deemed to be inconclusive.

**Table 1:** Distribution of secretor status among mother and children enrolled at the MAL-ED Bangladesh site

	Secretor status					
	Secretor		Nonsecretor		Inconclusive	
Children (*n*, %)	108 (58.7%)		48 (26.1%)		28 (15.2%)	
	Male	Female	Male	Female	Male	Female
	54 (50%)	54 (50%)	26 (54.2%)	22 (45.8%)	15 (53.6%)	13 (46.4%)
Mother (*n*, %)	101 (64.7%)		35 (22.4%)		16 (10.3%)	

### Sociodemographic characteristics of the study participants based on their secretor status.

We also analyzed the sociodemographic characteristics of the study participants based on their secretor status (Table 2). The percentage of male children was higher among the nonsecretors compared with the secretors (56.5% versus 50%). Additionally, the mothers of the secretor children were significantly less uneducated compared with the mothers of the nonsecretor children (*P* = 0.04). The monthly family income was also significantly lower among the secretor children (*P* < 0.01). Moreover, the percentage of secretor mothers among the secretor children (65.7%) was significantly higher (*P* < 0.01) than the percentage of secretor mothers among nonsecretor children (41.7%).

**Table 2:** Comparison of general characteristics of the study participants enrolled at the MAL-ED Bangladesh site on the basis of their secretor status

Sociodemographic characteristics	Secretor (*N* = 108)	Nonsecretor (*N* = 48)	*P* value
Male sex*	54 (50%)	26 (54.2%)	0.428
Days of exclusive breastfeeding†	146.3 ± 41.6	146.9 ± 36.2	0.464
Birth weight (kg)†	2.77 ± 0.43	2.82 ± 0.40	0.295
Weight-for-age *z* score at enrollment†	−1.36 ± 0.97	−1.32 ± 0.89	0.408
Length-for-age *z* score at enrollment†	−1.2 ± 1.11	−0.98 ± 0.86	0.260
Length-for-age *z* score at 24 months†	−2.20 ± 0.95	−2.06 ± 0.89	0.205
Weight-for-age *z* score at 24 months†	−1.82 ± 0.89	−1.67 ± 0.96	0.176
Maternal age (years)†	25 ± 5.06	24.5 ± 495	0.725
Maternal weight (kg)†	48.1 ± 6.96	50 ± 8.92	0.904
Maternal height (cm)†	148.9 ± 5.19	149.3 ± 4.51	0.368
Maternal educational level < 6 years*	69 (63.9%)	24 (50%)	0.04
Mother has less than three living children*	81 (75%)	39 (81.3%)	0.285
Routine treatment of drinking water*	68 (63%)	29 (60.4%)	0.839
Monthly income < $150*	25 (23.2%)	22 (45.8%)	< 0.01
Secretor mother*	71 (65.7%)	20 (41.7%)	< 0.01

* Data represented as *n*, %.

† Data represented as mean ± SD.

### Distribution of the enteric inflammatory biomarkers between the secretors and nonsecretors from 1 to 24 months of age.

**Figure 1.**
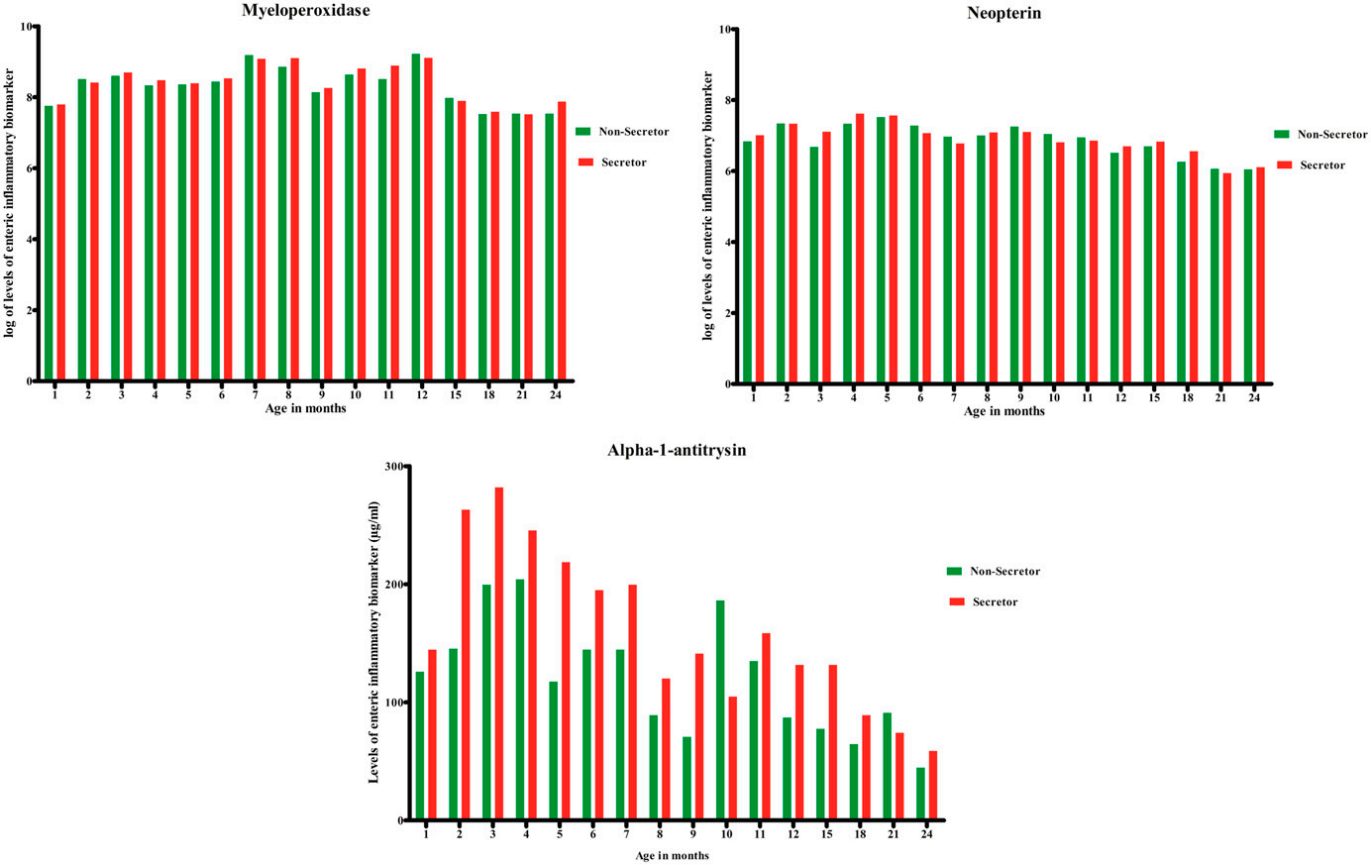
Distribution of the enteric inflammatory biomarkers between the secretors and nonsecretors from 1 to 24 months of age. This figure appears in color at www.ajtmh.org.

Figure 1 shows the distribution of the levels of the enteric inflammatory biomarkers, namely MPO, NEO, and AAT among the secretors and nonsecretors from 1 to 24 months of age. In general, the levels of MPO and NEO were greater than that of AAT at all time intervals among both the secretors and nonsecretors. When compared between the secretors and nonsecretors, the levels of MPO and NEO remained comparable between the secretors and the nonsecretors. However, the levels of AAT were found to be higher among the secretors at each of the time intervals, except for month 10.

### Association of secretor status of children with childhood enteropathy and childhood growth.

Table 3 indicates the results of the multivariate GEE analysis showing association of the secretor status of the study participants with childhood enteropathy, as denoted by the enteric inflammatory biomarkers: MPO, NEO, and AAT, as well as the EED score. After adjusting for relevant covariates including sex of the child, age of the child, birth weight, duration of exclusive breastfeeding, maternal age, WAZ at enrolment, WAMI, and detection of enteropathogens associated with the secretor status of children, we found a significant positive association between AAT levels and the EED score with the secretor-positive status of the children. Our findings indicate that among the secretor children the AAT levels and the EED scores are likely to be greater than that among nonsecretor children by 0.11 units (*P* < 0.01) and 0.32 units (*P* = 0.05), respectively. However, we did not find any significant association between MPO levels and the NEO levels with the secretor-positive status of children, despite having a positive effect size (as represented by the positive β-coefficient) for the associations of MPO and NEO with secretor-positive status of children. On the other hand, we did not find any significant association between secretor-positive status of children with the anthropometric indices of LAZ and WAZ, respectively (Table 4). Nevertheless, we observed a negative effect size (as represented by the negative β-coefficient) for the association between secretor-positive status of children with LAZ and WAZ, respectively, indicating a decreasing trend of LAZ and WAZ among secretor-positive children.

**Table 3:** Association of enteric inflammation with the secretor status of the participants enrolled at the MAL-ED Bangladesh site

Explanatory variable	Myeloperoxidase		Neopterin		Alpha-1-antitrypsin		EED score	
	Adjusted β-coefficient (95% CI)	*P* value	Adjusted β-coefficient (95% CI)	*P* value	Adjusted β-coefficient (95% CI)	*P* value	Adjusted β-coefficient (95% CI)	*P* value
Secretor child	0.04 (−0.07, 0.10)	0.21	0.06 (−0.09, 0.20)	0.13	0.11 (0.07, 0.21)	< 0.01	0.32 (0.29, 0.65)	0.05
Female sex	0.06 (−0.07, 0.19)	0.16	−0.08 (−0.07, 0.06)	0.15	0.19 (0.05, 0.22)	0.04	0.27 (0.04, 0.58)	0.09
Age of child in months	−0.03 (−0.04, −0.02)	< 0.01	−0.06 (−0.07, −0.04)	< 0.01	−0.18 (−0.33, −0.13)	< 0.01	−0.11 (−0.13, −0.09)	< 0.01
Birth weight in kg	0.17 (−0.04, 0.38)	0.19	−0.07 (−0.30, 0.16)	0.52	0.29 (−0.14, 0.4)	0.24	0.19 (0.04, 0.27)	0.15
Duration of exclusive breastfeeding in days	−0.01 (−0.14, 0.11)	0.17	−0.2 (−0.4, −0.05)	0.01	0.03 (0.01, 0.05)	< 0.01	−0.06 (−0.09, −0.03)	< 0.01
Maternal age in years	−0.02 (−0.15, 0.10)	0.13	−0.03 (−0.16, 0.11)	0.12	−0.01 (−0.02, 0.05)	0.22	−0.16 (−0.46, 0.02)	0.11
Length-for-age *z* score at enrollment	0.09 (−0.08, 0.09)	0.14	0.06 (−0.03, 0.15)	0.18	0.07 (0.03, 0.14)	0.04	0.17 (−0.03, 0.37)	0.09
Detection of Norovirus genogroup II	0.16 (0.03, 0.36)	0.05	−0.05 (−0.24, 0.13)	0.12	0.04 (−0.06, 0.16)	0.10	0.23 (−0.08, 0.55)	0.15
Detection of Rotavirus	−0.025 (−0.26, 0.02)	0.13	0.27 (0.15, 0.55)	0.03	0.13 (0.03, 0.29)	0.01	0.40 (0.86, 0.27)	0.09
Detection of *Campylobacter jejuni/coli*	0.22 (0.06, 0.39)	< 0.01	−0.03 (−0.19, 0.13)	0.29	0.03 (−0.03, 0.07)	0.19	0.10 (0.05, 0.20)	0.04
Detection of typical EPEC	−0.02 (−0.13, 0.01)	0.10	0.19 (0.04, 0.37)	0.04	−0.07 (−0.17, 0.03)	0.11	0.46 (0.92, 0.40)	0.03
Detection of *Cryptosporidium* sp.	0.15 (0.13, 0.33)	0.04	0.25 (0.19, 0.53)	< 0.01	0.08 (0.04, 0.18)	0.04	0.57 (0.23, 0.91)	< 0.01
Detection of *Giardia* sp.	0.36 (0.19, 0.53)	< 0.01	0.10 (−0.29, 0.20)	0.31	0.12 (0.06, 0.24)	0.04	0.37 (0.31, 1.05)	0.03
Detection of *Helicobacter pylori*	0.20 (−0.14, 0.53)	0.25	0.36 (0.29, 0.74)	<0.01	0.30 (0.25, 0.54)	0.04	−0.05 (−0.18, 0.29)	0.65
Detection of ETEC	0.21 (0.08, 0.32)	< 0.01	−0.02 (−0.16, 0.12)	0.21	0.12 (0.04, 0.17)	0.02	0.55 (−1.05, 0.89)	0.26

EED = environmental enteric dysfunction.

**Table 4:** Association of length-for-age and weight-for-age *z* scores with the secretor status of the participants enrolled at the MAL-ED Bangladesh site

Explanatory variables	Length-for-age *z* score		Weight-for-age *z* score	
	Adjusted β-coefficient (95% CI)	*P* value	Adjusted β-coefficient (95% CI)	*P* value
Secretor child	−0.14 (−0.24, 0.11)	0.18	−0.23 (−0.35, 0.07)	0.13
Female sex	0.28 (0.04, 0.51)	0.02	−0.04 (−0.27, 0.25)	0.27
Age of child in months	−0.06 (−0.07, −0.04)	< 0.01	−0.04 (−0.09, −0.03)	< 0.01
Birth weight in kg	0.58 (0.18, 1.17)	0.03	0.64 (0.19, 1.09)	< 0.01
WAMI score	0.36 (0.06, 0.60)	0.05	0.27 (−0.01, 0.48)	0.36
Maternal height in cm	0.13 (−0.02, 0.40)	0.17	0.18 (−0.01, 0.24)	0.23
Maternal weight in kg	0.19 (0.7, 0.29)	0.02	0.16 (0.12, 0.26)	< 0.01
Length-for-age *z* score at enrollment	0.50 (0.30, 0.69)	< 0.01	0.07 (−0.12, 0.26)	0.25
Detection of EAEC	−0.34 (−0.69, −0.11)	0.04	−0.30 (−0.38, −0.32)	< 0.01
Detection of *Campylobacter jejuni/coli*	0.021 (−0.02, 0.06)	0.19	0.02 (−0.08, 0.05)	0.22
Detection of ST-ETEC	−0.12 (−0.26, −0.06)	0.03	−0.36 (−0.54, −0.21)	< 0.01
Detection of *Shigella* sp.	−0.11 (−0.41, −0.06)	0.02	−0.37 (−0.71, −0.14)	< 0.01
Detection of Norovirus genogroup I	0.05 (−0.03, 0.07)	0.21	−0.39 (−0.41, −0.32)	< 0.01
Detection of *Giardia* sp.	−0.14 (−0.49, −0.08)	0.01	0.11 (−0.03, 0.15)	0.15
Secretor mother	−0.21 (−0.38, −0.07)	0.02	−0.09 (−0.41. 0.02)	0.10

WAMI = Water, sanitation, hygiene, Asset, Maternal education, and Income index.

## DISCUSSION

Our study findings show the distribution of secretor status among Bangladeshi children and their mothers and illustrate a significant positive association between childhood enteropathy (as denoted by the EED score) and secretor-positive status of Bangladeshi children. Previous findings have shown that the prevalence of the secretor phenotype varies with different ethnicities, with a prevalence of 70–80% among Caucasians but only 55% among Africans.^46^^,^^47^ On the other hand, a number of concurrent studies have demonstrated that the prevalence of Lewis-negative individuals, expressing neither Lewis A nor Lewis B and classified as “inconclusive secretor status” is approximately 7%.^48^^–^^50^ In our study, we found that the prevalence of secretors was approximately 59% among the children and 65% among the mothers. This observation differs from the reports from an observational study conducted among voluntary blood donors in Tamil Nadu, India, where they found that the prevalence of secretors was 75%.^51^ In our study, we report higher nonsecretor prevalence among males. In a study in Manipur, India, 10% higher nonsecretor rate was found among males^52^ and similar results were found in Rome among asthmatic patients.^53^ Additionally, we also report approximately 15% and 10% prevalence of “inconclusive secretor status/Lewis negative” among the children and mothers, respectively. This is in concordance with the findings from the previously mentioned observational study conducted among voluntary blood donors in India, where they reported a 15% prevalence of Lewis-negative individuals.^51^

Moreover, we report that the prevalence of secretor mothers was more among the secretor children and vice versa for nonsecretors (Table 2). This phenomenon may be explained by the presence of Lewis antigens in breast milk,^54^ which in turn may act as inducers for the activation of genes responsible for the expression of Lewis antigens in children. In addition, the maternal secretor status influences the distribution and concentration of human milk oligosaccharides (HMOs) expressed in breast milk. These changes in breast milk composition may result in alteration of the microbiome of the child,^55^^,^^56^ which in turn may lead to a difference in the innate immune system of the child for resistance to enteric infections. The HMOs are modified in secretor mothers’ milk making the milk richer in fucosylated oligosaccharides. It is speculated that these modified HMOs can act as decoy receptors serving as anti-adhesive antimicrobials, thus, protecting breastfed babies from enteric infections. Maternal secretor status may also act through its effect on a child’s gut microbiota. The digestion-resistant HMOs serve as metabolic substrates for intestinal microbiota and help shape microbiota composition.^57^^–^^59^

Children who are secretors present Lewis antigens on the lining of their gut epithelium, which, in turn, acts as attachment sites or “decoys” for the binding of a plethora of enteropathogens, before host cell invasion and pathogenesis.^60^^–^^63^ A previous study conducted in Burkina Faso reported that nonsecretors were protected against infections by certain genotypes of rotavirus.^64^ Recent findings from a multicenter study showed strong associations between Lewis group antigens and enteric infection burden for some specific enteropathogens.^37^ Henceforth, secretor children are more susceptible to enteric inflammation.^60^^–^^63^ This is evidenced by our study findings, whereby we demonstrate a significant positive association between secretor-positive status and AAT and EED score (Table 3). The respective effect size for the association of the outcome variable of AAT and EED score and the enteropathogens that have been previously reported to be associated with the secretor status of children have also been shown in Table 3. Our results thus indicate that secretor-positive children are at greater risk of development of enteric inflammation and concurrent protein loss, which may reflect the negative effect size for the association of secretor-positive status of children with their LAZ and WAZ, respectively, despite not being statistically significant (Table 4).

The strengths of our study involve the use of a sophisticated and extensively validated multiplex quantitative PCR (qPCR) system for the detection of a number of enteropathogens from a single sample and that we were able to include both the children and their mothers for the assessment of secretor status and its association with childhood enteropathy. However, we were not able to assess the secretor status for the mothers of all the children, whom we had enrolled in our study. We were unable to do genotyping for the individuals (both children and mothers) for whom we were unable to determine the secretor status by using the in-house ELISA method. Henceforth, the possibility of novel polymorphisms existing in the FUT-2 and FUT-3 genes of these individuals with “inconclusive secretor status” is thus very much pertinent with regard to the limitations of our study. In addition, small intestinal endoscopy, the gold standard for diagnosis of EED, was not performed and so we do not have any pertinent data regarding any possible molecular or immunological aberrations in the upper GI biopsy specimens of the study children.

## Conclusion

From our study, we report that the prevalence of secretors among Bangladesh children was approximately 59% and that among their mothers was 65%. The percentage of males among the nonsecretors was greater than that of the females in the nonsecretor group. Our study findings show a potential positive association between the secretor-positive status of Bangladeshi children with childhood enteropathy. Because enteropathy is considered to be an integral factor for childhood growth failure. Our findings thus implicate toward the genetic factor of secretor status of children being associated being linked with childhood growth faltering, through increased susceptibility to distinct enteropathogens and the consequent development of enteric inflammation and enteropathy among children. Thus, our study findings provide unprecedented cues responsible for enteropathy among children and the consequent growth failure. However, our findings are only applicable to settings in Bangladesh and thus need to be validated in several other similar settings, to establish a possible relationship between the secretor status of children with enteropathy and eventual childhood growth failure.

## References

[1] Keusch GT 2014. Environmental enteric dysfunction: pathogenesis, diagnosis, and clinical consequences. Clin Infect Dis 59 (Suppl_4): S207–S212. 25305288 10.1093/cid/ciu485 PMC4481570

[2] Kosek MN , MAL-ED Network Investigators , 2017. Causal pathways from enteropathogens to environmental enteropathy: findings from the MAL-ED birth cohort study. EBioMedicine 18: 109–117. 28396264 10.1016/j.ebiom.2017.02.024 PMC5405169

[3] George CM 2018. Enteric infections in young children are associated with environmental enteropathy and impaired growth. Trop Med Int Health 23: 26–33. 29121442 10.1111/tmi.13002

[4] Chen RY 2021. A microbiota-directed food intervention for undernourished children. N Engl J Med 384: 1517–1528. 33826814 10.1056/NEJMoa2023294 PMC7993600

[5] Haque MA 2019. Determinants of *Campylobacter* infection and association with growth and enteric inflammation in children under 2 years of age in low-resource settings. Sci Rep 9: 1–8. 31748573 10.1038/s41598-019-53533-3 PMC6868199

[6] Das R Palit P Haque M Mahfuz M Faruque A Ahmed T , 2021. Site specific incidence rate of virulence related genes of enteroaggregative *Escherichia coli* and association with enteric inflammation and growth in children. Sci Rep 11: 1–10. 34848801 10.1038/s41598-021-02626-z PMC8632913

[7] Palit P Das R Haque A Nuzhat S Khan SS Siddiqua TJ Mahfuz M Faruque ASG Ahmed T , 2022. Risk factors for enterotoxigenic bacteroides fragilis infection and association with environmental enteric dysfunction and linear growth in children: results from the MAL-ED study. Am J Trop Med Hyg 106: 915. 35100563 10.4269/ajtmh.21-0780 PMC8922507

[8] George CM 2015. Fecal markers of environmental enteropathy are associated with animal exposure and caregiver hygiene in Bangladesh. Am J Trop Med Hyg 93: 269–275. 26055734 10.4269/ajtmh.14-0694 PMC4530746

[9] Mahfuz M 2017. Bangladesh Environmental Enteric Dysfunction (BEED) study: protocol for a community-based intervention study to validate non-invasive biomarkers of environmental enteric dysfunction. BMJ Open 7: e017768. 10.1136/bmjopen-2017-017768 PMC5724211 28801442

[10] Iqbal NT 2019. Study of Environmental Enteropathy and Malnutrition (SEEM) in Pakistan: protocols for biopsy based biomarker discovery and validation. BMC Pediatr 19: 1–17. 31331393 10.1186/s12887-019-1564-x PMC6643315

[11] McCormick BJ 2017. Dynamics and trends in fecal biomarkers of gut function in children from 1–24 months in the MAL-ED study. Am J Trop Med Hyg 96: 465–472. 27994110 10.4269/ajtmh.16-0496 PMC5303054

[12] Iqbal NT 2018. Promising biomarkers of environmental enteric dysfunction: a prospective cohort study in Pakistani children. Sci Rep 8: 1–10. 29445110 10.1038/s41598-018-21319-8 PMC5813024

[13] Fahim SM Das S Sanin KI Gazi A Mahfuz M Islam MM Ahmed T , 2018. Association of fecal markers of environmental enteric dysfunction with zinc and iron status among children at first two years of life in Bangladesh. Am J Trop Med Hyg 99: 489–494. 29893201 10.4269/ajtmh.17-0985 PMC6090336

[14] Ferrer-Admetlla A Sikora M Laayouni H Esteve A Roubinet F Blancher A Calafell F Bertranpetit J Casals F , 2009. A natural history of FUT_2_ polymorphism in humans. Mol Biol Evol 26: 1993–2003. 19487333 10.1093/molbev/msp108

[15] Marionneau S Ruvoën N Le Moullac-Vaidye B Clement M Cailleau-Thomas A Ruiz-Palacois G Huang P Jiang X Le Pendu J , 2002. Norwalk virus binds to histo-blood group antigens present on gastroduodenal epithelial cells of secretor individuals. Gastroenterology 122: 1967–1977. 12055602 10.1053/gast.2002.33661 PMC7172544

[16] Kelly RJ Rouquier S Giorgi D Lennon GG Lowe JB , 1995. Sequence and expression of a candidate for the human secretor blood group α (1, 2) fucosyltransferase gene (FUT2) homozygosity for an enzyme-inactivating nonsense mutation commonly correlates with the non-secretor phenotype. J Biol Chem 270: 4640–4649. 7876235 10.1074/jbc.270.9.4640

[17] Yamamoto F-I Clausen H White T Marken J Hakomori S-I , 1990. Molecular genetic basis of the histo-blood group ABO system. Nature 345: 229–233. 2333095 10.1038/345229a0

[18] Clausen H Hakomori S , 1989. ABH and related histo‐blood group antigens; Immunochemical differences in carrier isotypes and their distribution 1. Vox Sang 56: 1–20. 2464874 10.1111/j.1423-0410.1989.tb03040.x

[19] Daniels G 3rd , 2013. Human Blood Groups, 3rd edition. Oxford, United Kingdom: Blackwell Publishing.

[20] Henry S Oriol R Samuelsson B , 1995. Lewis histo‐blood group system and associated secretory phenotypes. Vox Sang 69: 166–182. 8578728 10.1111/j.1423-0410.1995.tb02591.x

[21] Sharma S Hagbom M Svensson L Nordgren J , 2020. The impact of human genetic polymorphisms on rotavirus susceptibility, epidemiology, and vaccine take. Viruses 12: 324. 10.3390/v12030324 PMC7150750 32192193

[22] Parker EP Ramani S Lopman BA Church JA Iturriza-Gómara M Prendergast AJ Grassly NC , 2018. Causes of impaired oral vaccine efficacy in developing countries. Future Microbiol 13: 97–118. 29218997 10.2217/fmb-2017-0128 PMC7026772

[23] Imbert-Marcille B-M Barbé L Dupé M Le Moullac-Vaidye B Besse B Peltier C Ruvoën-Clouet N Le Pendu J , 2014. A FUT2 gene common polymorphism determines resistance to rotavirus A of the P [8] genotype. J Infect Dis 209: 1227–1230. 24277741 10.1093/infdis/jit655

[24] Ansari SA Khan A Khan TA Raza Y Syed SA Akhtar SS Kazmi SU , 2015. Correlation of ABH blood group antigens secretion with *Helicobacter pylori* infection in Pakistani patients. Trop Med Int Health 20: 115–119. 25322664 10.1111/tmi.12401

[25] Azevedo M 2008. Infection by *Helicobacter pylori* expressing the BabA adhesin is influenced by the secretor phenotype. J Pathol 215: 308–316. 18498114 10.1002/path.2363

[26] Rausch P Rehman A Künzel S Häsler S Ott SJ Schreiber S Rosenstiel P Franke A Baines JF , 2011. Colonic mucosa-associated microbiota is influenced by an interaction of Crohn disease and FUT2 (Secretor) genotype. Proc Natl Acad Sci USA 108: 19030–19035. 22068912 10.1073/pnas.1106408108 PMC3223430

[27] Nordgren J Sharma S Kambhampati A Lopman B Svensson L , 2016. Innate resistance and susceptibility to norovirus infection. PLoS Pathog 12: e1005385. 27115484 10.1371/journal.ppat.1005385 PMC4845991

[28] Nordgren J Kindberg E Lindgren P-E Matussek A Svensson L , 2010. Norovirus gastroenteritis outbreak with a secretor-independent susceptibility pattern, Sweden. Emerg Infect Dis 16: 81. 20031047 10.3201/eid1601.090633 PMC2874438

[29] Payne DC 2015. Epidemiologic association between FUT2 secretor status and severe rotavirus gastroenteritis in children in the United States. JAMA Pediatr 169: 1040–1045. 26389824 10.1001/jamapediatrics.2015.2002 PMC4856001

[30] Ahmed T 2014. The MAL-ED cohort study in Mirpur, Bangladesh. Clin Infect Dis 59: S280–S286. 25305298 10.1093/cid/ciu458

[31] The MALEDNI , 2014. The MAL-ED Study: a multinational and multidisciplinary approach to understand the relationship between enteric pathogens, malnutrition, gut physiology, physical growth, cognitive development, and immune responses in infants and children up to 2 years of age in resource-poor environments. Clin Infect Dis 59 (Suppl_4): S193–S206. 25305287 10.1093/cid/ciu653

[32] Richard SA Barrett LJ Guerrant RL Checkley W Miller MA , 2014. Disease surveillance methods used in the 8-site MAL-ED cohort study. Clin Infect Dis 59 (Suppl_4): S220–S224. 25305290 10.1093/cid/ciu435 PMC4204606

[33] Das R Jobayer Chisti M Ahshanul Haque M Ashraful Alam M Das S Mahfuz M Mondal D Ahmed T, 2021. Evaluating association of vaccine response to low serum zinc and vitamin D levels in children of a birth cohort study in Dhaka. Vaccine 39: 59–67. 33121844 10.1016/j.vaccine.2020.10.048 PMC7735373

[34] Das S Alam MA Mahfuz M El Arifeen S Ahmed T , 2019. Relative contributions of the correlates of stunting in explaining the mean length-for-age z-score difference between 24-month-old stunted and non-stunted children living in a slum of Dhaka, Bangladesh: results from a decomposition analysis. BMJ Open 9: e025439. 10.1136/bmjopen-2018-025439 PMC6678062 31366637

[35] UNICEF, WHO 2012. *WHO Joint Monitoring Programme for Water Supply and Sanitation.* Progress on drinking water and sanitation.

[36] Amour C 2016. Epidemiology and impact of *Campylobacter* infection in children in 8 low-resource settings: results from the MAL-ED study. Clin Infect Dis 63: 1171–1179. 27501842 10.1093/cid/ciw542 PMC5064165

[37] Colston JM 2019. Effects of child and maternal histo-blood group antigen status on symptomatic and asymptomatic enteric infections in early childhood. J Infect Dis 220: 151–162. 30768135 10.1093/infdis/jiz072 PMC6548901

[38] Reeck A Kavanagh O Estes MK Opekun AR Gilger MA Graham DY Atmar RL , 2010. Serological correlate of protection against norovirus-induced gastroenteritis. J Infect Dis 202: 1212–1218. 20815703 10.1086/656364 PMC2945238

[39] Kosek M 2013. Fecal markers of intestinal inflammation and permeability associated with the subsequent acquisition of linear growth deficits in infants. Am J Trop Med Hyg 88: 390. 23185075 10.4269/ajtmh.2012.12-0549 PMC3583335

[40] Liu J 2014. Development and assessment of molecular diagnostic tests for 15 enteropathogens causing childhood diarrhoea: a multicentre study. Lancet Infect Dis 14: 716–724. 25022434 10.1016/S1473-3099(14)70808-4

[41] Rogawski ET 2018. Use of quantitative molecular diagnostic methods to investigate the effect of enteropathogen infections on linear growth in children in low-resource settings: longitudinal analysis of results from the MAL-ED cohort study. Lancet Glob Health 6: e1319–e1328. 30287125 10.1016/S2214-109X(18)30351-6 PMC6227248

[42] Investigators M-EN , 2017. Childhood stunting in relation to the pre-and postnatal environment during the first 2 years of life: the MAL-ED longitudinal birth cohort study. PLoS Med 14: e1002408. 29069076 10.1371/journal.pmed.1002408 PMC5656304

[43] Ngure FM Reid BM Humphrey JH Mbuya MN Pelto G Stoltzfus RJ , 2014. Water, Sanitation, and Hygiene (WASH), environmental enteropathy, nutrition, and early child development: making the links. Ann N Y Acad Sci 1308: 118–128. 24571214 10.1111/nyas.12330

[44] Islam MM Sanin KI Mahfuz M Shamsir Ahmed AM Mondal D Haque R Ahmed T , 2018. Risk factors of stunting among children living in an urban slum of Bangladesh: findings of a prospective cohort study. BMC Public Health 18: 1–13. 10.1186/s12889-018-5101-x PMC5789576 29378556

[45] Platts-Mills JA 2017. Association between enteropathogens and malnutrition in children aged 6–23 mo in Bangladesh: a case-control study. Am J Clin Nutr 105: 1132–1138. 28381477 10.3945/ajcn.116.138800 PMC5402031

[46] King JR Varadé J Hammarström L , 2019. Fucosyltransferase gene polymorphisms and Lewisb-negative status are frequent in Swedish newborns, with implications for infectious disease susceptibility and personalized medicine. J Pediatric Infect Dis Soc 8: 507–518. 30544260 10.1093/jpids/piy085

[47] Sharma S Nordgren J , 2021. Effect of infant and maternal secretor status on rotavirus vaccine take—an overview. Viruses 13: 1144. 34198720 10.3390/v13061144 PMC8232156

[48] Torrado J Ruiz B Garay J Cosme A Arenas JI Bravo JC Fontham E Correa P , 1997. Lewis, secretor, and ABO phenotypes, and sulfomucin expression in gastric intestinal metaplasia. Cancer Epidemiol Biomarkers Prev 6: 287–289. 9107434

[49] Serpa J 2003. Lewis enzyme (α1–3/4 fucosyltransferase) polymorphisms do not explain the Lewis phenotype in the gastric mucosa of a Portuguese population. J Hum Genet 48: 183–189. 12730721 10.1007/s10038-003-0007-5

[50] Larsson MM Rydell GEP Grahn A Rodriguez-Diaz J Akerlind B Hutson AM Estes MK Larson G Svensson L , 2006. Antibody prevalence and titer to norovirus (genogroup II) correlate with secretor (FUT2) but not with ABO phenotype or Lewis (FUT3) genotype. J Infect Dis 194: 1422–1427. 17054072 10.1086/508430

[51] Bharath RR Arumugam P , 2016. The prevalence of secretor status and co-expression of lewis antigen in voluntary blood donors. Asian J Med Sci 7: 93–96.

[52] MAL-ED Network of Investigators , 2014. The MAL-ED study: a multinational and multidisciplinary approach to understand the relationship between enteric pathogens, malnutrition, gut physiology, physical growth, cognitive development, and immune responses in infants and children up to 2 years of age in resource-poor environments. Clin Infect Dis 59 (Suppl 4): S193–S206. 25305287 10.1093/cid/ciu653

[53] Ronchetti F Villa MP Ronchetti R Bonci E Latini L Pascone R Bottini N Gloria-Bottini F , 2001. ABO/Secretor genetic complex and susceptibility to asthma in childhood. Eur Respir J 17: 1236–1238. 11491170 10.1183/09031936.01.99109101

[54] Andreas NJ Al-Khalidi A Jaiteh M Clarke E Hyde MJ Modi N Holmes E Kampmann B Le Doare KM , 2016. Role of human milk oligosaccharides in Group B *Streptococcus* colonisation. Clin Transl Immunology 5: e99. 27588204 10.1038/cti.2016.43 PMC5007626

[55] Liu F Yan J Wang X Wang C Chen L Li Y Chen J Guo H , 2021. Maternal fucosyltransferase 2 status associates with the profiles of human milk oligosaccharides and the fecal microbiota composition of breastfed infants. J Agric Food Chem 69: 3032–3043. 33677972 10.1021/acs.jafc.0c04575

[56] Zivkovic AM German JB Lebrilla CB Mills DA , 2011. Human milk glycobiome and its impact on the infant gastrointestinal microbiota. Proc Natl Acad Sci USA 108 (Suppl 1): 4653–4658. 20679197 10.1073/pnas.1000083107 PMC3063602

[57] Smith-Brown P, Morrison M, Krause L, Davies PS, 2016. Mothers secretor status affects development of childrens microbiota composition and function: a pilot study. *PLoS One 11:* p.e0161211. 10.1371/journal.pone.0161211 PMC5028039 27644050

[58] Davis JC Lewis ZT Krishnan S Bernstein RM Moore SE Prentice AM Mills DA Lebrilla CB Zivkovic AM , 2017. Growth and morbidity of Gambian infants are influenced by maternal milk oligosaccharides and infant gut microbiota. Sci Rep 7: 40466. 28079170 10.1038/srep40466 PMC5227965

[59] Bode L , 2012. Human milk oligosaccharides: every baby needs a sugar mama. Glycobiology 22: 1147–1162. 22513036 10.1093/glycob/cws074 PMC3406618

[60] Arifuzzaman M 2011. Individuals with Le (a+ b−) blood group have increased susceptibility to symptomatic vibrio cholerae O1 infection. PLoS Negl Trop Dis 5: e1413. 22216364 10.1371/journal.pntd.0001413 PMC3246451

[61] Huang P 2003. Noroviruses bind to human ABO, Lewis, and secretor histo-blood group antigens: identification of 4 distinct strain-specific patterns. J Infect Dis 188: 19–31. 12825167 10.1086/375742

[62] Tan M Jiang X , 2014. Histo-blood group antigens: a common niche for norovirus and rotavirus. Expert Rev Mol Med 16: e5. 24606759 10.1017/erm.2014.2 PMC12406300

[63] Jiang X Liu Y Tan M , 2017. Histo-blood group antigens as receptors for rotavirus, new understanding on rotavirus epidemiology and vaccine strategy: rotavirus host receptor and vaccine strategy. Emerg Microbes Infect 6: 1–8. 10.1038/emi.2017.30 PMC5457676 28400594

[64] Nordgren J 2014. Both Lewis and secretor status mediate susceptibility to rotavirus infections in a rotavirus genotype–dependent manner. Clin Infect Dis 59: 1567–1573. 25097083 10.1093/cid/ciu633 PMC4650770

